# Unlocking the Potential of Disulfidptosis‐Related LncRNAs in Lung Adenocarcinoma: A Promising Prognostic LncRNA Model for Survival and Immunotherapy Prediction

**DOI:** 10.1002/cam4.70337

**Published:** 2024-10-21

**Authors:** Xin Nie, Hong Ge, Kongming Wu, Ru Liu, Chunyu He

**Affiliations:** ^1^ Department of Radiation Oncology The Affiliated Cancer Hospital of Zhengzhou University & Henan Cancer Hospital Zhengzhou People's Republic of China; ^2^ Department of Oncology Tongji Hospital of Tongji Medical College, Huazhong University of Science and Technology Wuhan People's Republic of China

**Keywords:** CIBERSORT, disulfidptosis, ESTIMATE, lncRNA, lung adenocarcinoma, TMB

## Abstract

**Objective:**

Disulfidptosis was stimulated in high SLC7A11 expression cells starving to glucose. We attempted to identify disulfidptosis‐related lncRNAs (DRLs), built a prognostic model to predict survival, and analyzed the tumor microenvironment.

**Methods:**

The TCGA database was utilized to procure the pertinent data. By utilizing both Cox regression and the least absolute shrinkage and selection operator (LASSO) method, a risk model based on DRLs was formulated for prognostic evaluation. The ability of survival prediction was validated by multiple approaches. The biological functions were screened through GO, KEGG, and GSEA. Various methods were employed to evaluate the tumor immune environment, which included ESTIMATE, tumor mutation burden (TMB) score, CIBERSORT algorithm, and tumor immune dysfunction and exclusion (TIDE) score.

**Results:**

Ninety‐one DRLs were recognized, and lncRNA *AC092718.4*, *AL365181.2*, *AL606489.1*, *EMSLR*, and *ENTPD3‐AS1* were involved in the risk model. The GEO database was used to verify the influence of these lncRNAs on survival. The following analyses showed that survival could be predicted excellently by the DRLs risk model. The results of enrichment analyses pointed toward the involvement of the cell cycle and IgA production pathways. In the low‐risk patient group, there was a notable surge in stromal, immune, and ESTIMATE scores, while the TMB scores took a tumble. Conversely, the high‐risk patient group displayed a converse trend. Notably, the group of patients with lower risk scores and higher TMB scores showed the most favorable survival outcomes, underscoring the importance of considering both risk score and TMB in predicting the response to immune checkpoint blockade therapy. Furthermore, patients classified as high‐risk might display resistance to both chemotherapy and targeted therapy. Cellular biological experiments proved that lncRNA *AC092718.4* promoted invasion, migration, and proliferation abilities in vitro. These results provided valuable insights into the role of DRLs in LUAD and presented a possible effective treatment approach for LUAD.

**Conclusions:**

We developed a disulfidptosis‐related risk model with 5 lncRNAs that enables survival prediciton for LUAD patients and aids cilinical decisions by forecasting the TME, TMB, and drug sensitivity, making it a valuable tool for outcomes prediction.

## 1 Introduction

Recently, a study from MD Anderson Cancer Center detailed disulfidptosis as a form of programmed cell death, which occurred due to disulfide bond formation in the cytoskeleton actins, which would be stimulated in high SLC7A11 expression cells starveling with glucose. In vitro experiments, disulfidptosis triggered by the glucose inhibitors could promote tumor extinction meanwhile the normal tissues had nice tolerance [1]. In clinical practice, the survival and recurrence patterns of patients could have been vastly different, even when they shared the same stage diagnosis of lung adenocarcinoma (LUAD) and treatment options, which suggested that other predication signatures rather than the clinical characteristics were urgently needed to achieve a precision individualized treatment strategy. Meanwhile, results of the online database indicated that disulfidptosis regulator SLC7A11 was overexpressed in lung cancer samples as well as cell lines [2]. Therefore, exploring the role of disulfidptosis has much potential for clinical utilization in lung cancer. Nowadays, prediction models of tumor risk score are a noninvasive tool for assessing patient survival [3], which could contribute to predicting prognosis and differentiating between patients who could potentially gain from treatment.

Previous studies implied that long noncoding RNAs (lncRNAs) participated in multiple physiological functions in lung cancer such as apoptosis, tumor microenvironment remodeling, drug resistance, and metastasis, and thus contribute to tumor progression and prognosis [4, 5, 6]. However, the potential role of disulfidptosis‐related lncRNAs (DRLs) in the progression of LUAD has not been elucidated in the previous studies thus far, and hence, there is a pressing need for further investigation in this regard.

In the current study, we built a 5‐DRLs risk core model trying to predict survival in TCGA‐LUAD samples for the first time and further explored the involved biological functions and signal pathways. Considering the increasing awareness about the significance of tumor microenvironment cells in the prognosis of lung cancer, we incorporated sophisticated tools to accurately estimate the presence of stromal and immune cells in the tumor microenvironment. To accomplish this, Estimation of STromal and Immune cells in MAlignant Tumor tissues using Expression (ESTIMATE) data [7] and CIBERSORT [8] algorithm were recruited to help us better understand the relationships in the prognostic DRLs, tumor microenvironment, and prognosis of LUAD. Finally, the capability of the risk model constructed from the five prognostic DRLs to predict drug sensitivity was explored in commonly used chemotherapy drugs and potential targeted therapy drugs. Our analysis emphasized the significance of our mode based on disulfidptosis related‐lncRNAs, laying the foundation for the therapeutic application of disulfidptosis.

## 2 Material and Methods

### 2.1 Data Download and Preparation

The datasets for this study were sourced from The Cancer Genome Atlas (TCGA) website (https://www.cancer.gov/ccg/research/genome‐sequencing/tcga), which provided the LUAD transcriptome RNA‐seq (STAR‐Counts), clinical, and simple nucleotide variation datasets. We gathered a total of 497 samples that were used for the analysis. To annotate the downloaded data, we utilized the annotation file from the GENCODE website (https://www.gencodegenes.org), which allowed for the identification of 16876 lncRNAs. And to validate the results of this study, GSE29013; GSE30219, GSE31210, AND GSE37745 were recruited. The use of these standardized and publicly available resources ensured the reliability and reproducibility of our results.

### 2.2 Identification of Disulfidptosis‐Related lncRNA

According to Liu et al. [1], the guide RNAs GYS1, LRPPRC, NCKAP1, NDUFA11, NDUFS1, NUBPL, *OXSM*, *RPN1*, *SLC3A2*, and *SLC7A1* were relatively fold changed between the glucose‐replete and ‐starved groups, and these 10 RNAs were adopted in our study as disulfidptosis‐related genes for further analyses. The DRLs were identified using the Pearson correlation with a cor‐filter ≥ 0.4 and *p* < 0.001. Then, 91 lncRNAs were identified to be co‐expressed with the DRGs and retrieved for the next analyses. The limma, dplyr, ggalluvial, and ggplot2 R packages were recruited for analyses and visualization in this section.

### 2.3 Establishment and Assessment of the 5‐DRL Risk Model of LUAD

Firstly, the univariable regression algorithm was applied to evaluate the candidate prognostic DRLs. Then, the LASSO and SVM‐RFE algorithms were applied to select prognostic lncRNAs from the candidate DRLs [9, 10]. By employing the multivariate Cox regression, a comprehensive risk model comprising the prognostic lncRNAs and the relevant correlation factors was derived. Then, we analyzed the correlation between the DRGs and prognostic DRLs using the relevant package. The process of classification of the high‐ and low‐risk groups was executed through the use of the median risk score from the 5‐DRLs signature model. To validate the model's ability to predict survival, a rigorous analysis was conducted, whereby all patient samples were randomly divided into training and test groups. Employing the Kaplan–Meier regression method and the receiver operating characteristic (ROC) curves coupled with the concordance index (C‐index) provided precise and detailed validation of the robustness of the risk model.

### 2.4 Construction of the Predictive Nomogram

We constructed a nomogram of survival prediction based on the risk score and the clinical parameters in the whole TCGA‐LUAD cohort. The nomogram could be used for evaluating the time‐dependent predictive efficacy of the overall survival rates at 1, 3, and 5 year. Following the development of the predictive nomogram, we developed a calibration curve to demonstrate the nomogram model's prediction ability.

### 2.5 Principal Component Analysis

Usually, the dimensional features could be reduced by the principal component analysis (PCA), which is a normally used statistical technique. Thus, we applied the scatterplot3d R package to conduct PCA and judge the classification capability of varied gene clusters.

### 2.6 Implementation of Tumor Immune Microenvironment Analysis and Prediction of Drug Sensitivity

The relationship in the risk score, tumor mutant burden, and the tumor immune microenvironment was evaluated by the maftools R package, ESTIMATE R package, and CIBERSORT R script v1.03, respectively. The tumor immune dysfunction and exclusion (TIDE) analysis was carried out by uploading the expression matrix online (http://tide.dfci.harvard.edu/), and then the results for response prediction were downloaded for the following analyses. Finally, the sensitivities of chemotherapy and potential targeted therapy drugs were evaluated using data from the Genomics of Drug Sensitivity in Cancer (GDSC, https://www.cancerrxgene.org/) database and the oncoPredict R package.

### 2.7 Function Enrichment Analyses

Comparative analysis of gene expression between low‐ and high‐risk groups assessed different expression genes (DEGs), and the Gene Ontology (GO) analysis provided valuable insights into the molecular functions, cellular components, and biological roles that were enriched in the DEGs. Meanwhile, the Kyoto Encyclopedia of Genes and Genomes (KEGG) analysis enabled us to identify the specific signaling pathways that were significantly affected by these DEGs. Going a step further, instead of concentrating on DEGs in the above analyses, Gene set enrichment analysis (GSEA) sorts all the genes according to their expression and then gives each gene an ES enrichment score. Thus, GSEA was also applied for further understanding of the involved functions and signaling pathways.

### 2.8 Cell Culture

Human lung epithelial BEAS‐2B cells, human lung adenocarcinoma cell line NCI‐H1975, NCI‐1299, and human large cell lung cancer cell line NCI‐H460 were purchased from the Procell Life Science & Technology (Wuhan, China) and were cultured in DMEM and RPMI‐1640 medium (KeyGEN BioTECH, China) containing 10% fetal bovine serum (Gibco, USA) and 1% Penicillin–Streptomycin (KeyGEN BioTECH, China) at 37°C in 5% CO_2_.

### 2.9 RNA Extraction and Quantitative Real‐Time PCR (qRT‐PCR)

The total RNA of BEAS‐2B, NCI‐H1975, NCI‐H1299, and NCI‐H460 cells was extracted using the RNA extraction kit (Beyotime, China) and reverse‐transcribed into cDNA using the HiScript II Q SuperMix for qPCR (Vazyme, China) following the users' manual. Then, the qRT‐PCR was carried out using the Taq Pro Universal SYBR qPCR Master Mix (Vazyme, China). The primer sequences were as follows: GAPDH‐forward: TGT ACG CCA ACA CAG TGC TG, GAPDH‐reverse: TCA GGAGGA GCA ATG ATC TTG, AL563181.2‐forward: CCC AAG AAC CTT AAC CAC CTC A, AL5631812‐reverse: CGT CAC TGG TCT GGC TTT CA, EMSLR‐forward: GTT TCC ACC TAG GAC TAC AGG CT, EMSLR‐reverse: CCC CGC CGA TCC AAT TTC TC, AC092718.4‐forward: TGG AGG GAG GAA GCC ATT CT, AC092718.4‐reverse: GGT TGG TGC TGT TGA GGA GT, ENTPD3‐AS1‐forward: AA GAC ACT TGA GGG GGA GGT, ENTPD3‐AS1‐reverse: GGG TCT CGC TAA CAC CAG AG, AL606489.1‐forward: AGG AAA GAC ATC AGC AGA GAG C, and AL606489.1‐reverse: GTA GCT GGG TGG GTG CAT TC. The ΔΔCq method was applied to calculate the relative lncRNA expression.

### 2.10 Transient Transfections

The vector and pcDNA3.1‐AC092718.4 were purchased from OBiO Technology (Shanghai, China). NCI‐H1975 and NCI‐H1299 cells were seeded in 6‐well plates at a count of 1 × 10^5^ and then cultured overnight. The next day, the mix of 2.5 μg of plasmid DNA (vector or pcDNA3.1‐AC092718.4), Opti‐MEM Medium (Gibco, USA), and Lipo8000 (Beyotime, China) was added into the 6‐well plates, which was prepared according to the users' manual. Then the medium was replaced by the fresh complete medium 24 h after transfection. The RNA was extracted 48 h after transfection and the relative expression of lncRNA *AC092718.4* was analyzed by qRT‐PCR.

### 2.11 Colony‐Forming Assay

The effect of lnc‐AC092718.4 on the proliferation of LUAD cells was analyzed by the colony‐forming assay. NCI‐H1975 and NCI‐H1299 cells transfected with vector or pcDNA3.1‐AC092718.4 were seeded in triplicate at a density of 4 × 10^3^ cells/well in 6‐well plates. After 7 days of culturing in a 37°C incubator with 5% CO2, the medium was removed. Then, the colonies were fixed with 4% polyformaldehyde and stained with 0.5% crystal violet. The plates were photographed using a smartphone and quantified using ImageJ.

### 2.12 Transwell Assays

The transwell chambers coated with or without Matrigel (Sigma, USA) were applied to explore the invasive ability or migration ability in the H1975 and H1299 cells, respectively. Briefly, cells were cultured in the serum‐free RPMI‐1640 for 24 h before the assay, then the Matrigel was diluted proportionally with precooled serum‐free 1640 medium. The transwell chambers coated with diluted Matrigel were used for detecting the invasive ability, while untreated chambers were used to assay migratory ability. Then the starvation preconditioning cells were counted and seeded in the upper chamber at a count of 1 × 10^5^. RPMI‐1640 medium containing 10% serum was added into the lower chamber and the plates were incubated for 24 h. Finally, Matrigel on the upper side of the insert was removed, and the cells on the lower side were fixed in methanol and stained using the crystal violet solution. The stained cells were counted as invasive/migrative cells.

### 2.13 CCK8 Assay

The proliferation ability was also detected by CCK8 assay. Briefly, cells were cultured in 1 × 10^3^ quantities in 96‐well plates, and the cell viability was assessed per 24 h over 96 h according to the users' manual of the CCK8 kit (Abbkine, China).

### 2.14 Statistical Analysis

We utilized the R software (version 4.2.2) as our primary analytical tool to perform all necessary statistical analyses. The relevant R packages (limma, dplyr, survival, ggalluvial, ggplot2, care, glmnet, survminer, timeROC, tidyverse, ggExtra, pheatmap, rms, pec, regplot, survcomp, scatterplot3d, colorspace, stringi, circlize, RColorBrewer, ggpubr, org.Hs.eg.db, enrichplot, estimate, clusterProfiler, reshape2, e1071, maftools, oncoPredict, and parallel) were recruited during the analyses. Statistical significance was set at *p* < 0.05.

## 3 Results

### 3.1 Identification of Disulfidptosis‐Related Long Noncoding RNAs and Construction of the Risk Model

**FIGURE 1:**
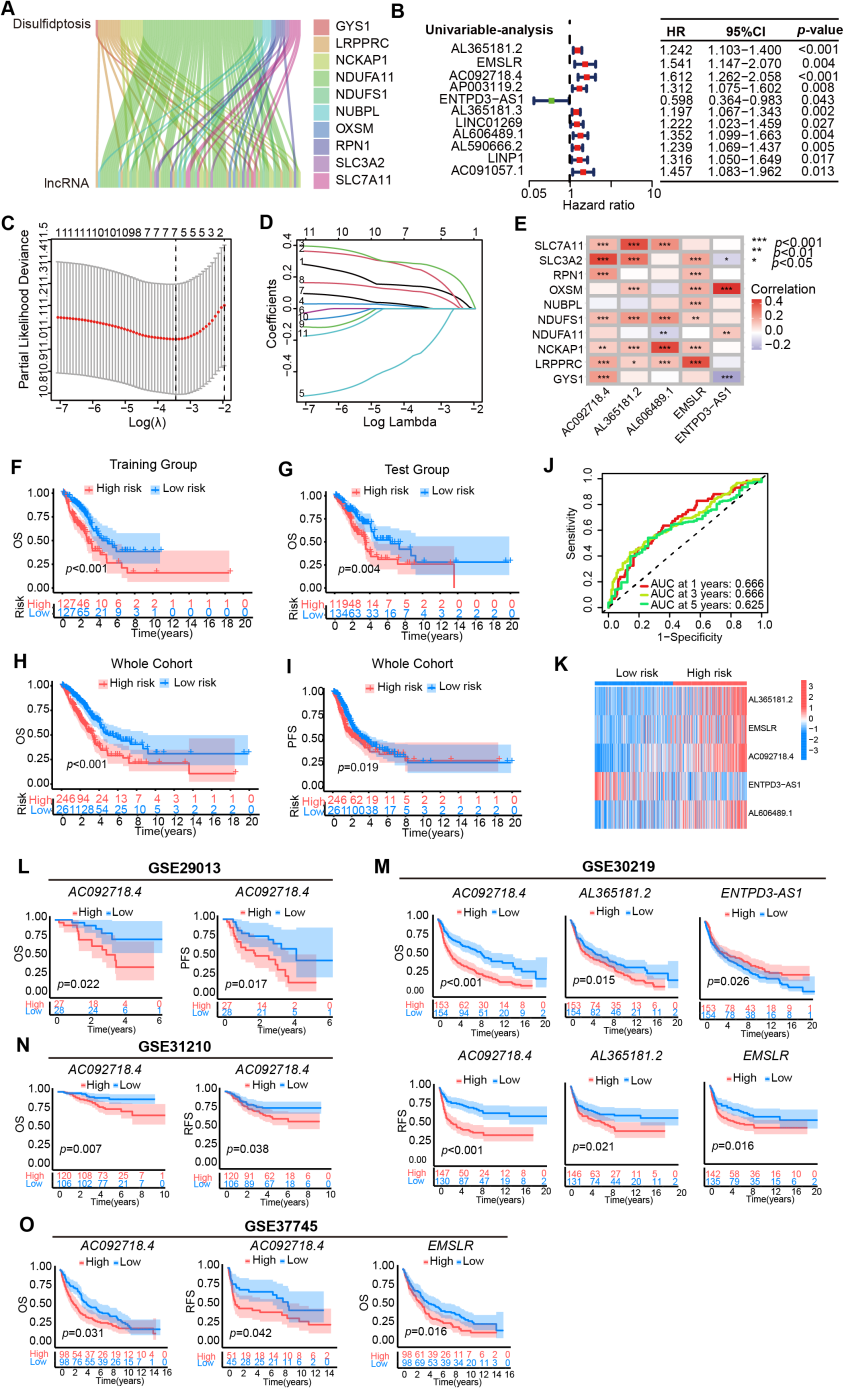
Identification and validation of the disulfidptosis‐related lncRNA risk model (DRLs) in the TCGA‐LUAD and GEO datasets. (A) The Sankey diagram showed that a total of 91 lncRNAs were co‐expressed with disulfidptosis‐related genes. (B) Univariable Cox regression was applied to assess the prognosis value of candidate DRLs. (C) Selection of adjusted parameters in the LASSO model using 10‐fold cross‐validation. (D) LASSO coefficient profiles of prognostic DRLs. (E) Heatmap indicating the correlation between the five prognostic DRLs and 10 DRGs. Kaplan–Meier curves for overall survival (OS) in training (F) and test (G) groups. (J) Time‐dependent ROC curves of OS at 1, 3, and 5 year, the sensitivities were 0.625, 0.666, and 0.666, respectively. (H) The OS and (I) progression‐free survival (PFS) differences are shown by Kaplan–Meier curves. (K) The expression of prognostic DRLs in low‐ and high‐risk groups. (L) High expression of *AC092718.4* was related to poor OS and PFS of patients in GSE29013. (M) High expression of *AC092718.4*, *AL365181.2*, *ENTPD3‐AS1*, or *EMSLR* was related to poor OS and recurrence‐free survival (RFS) in GSE30219. (N) High expression of *AC092718.4* was related to poor OS and RFS in GSE31210. (O) High expression of *AC092718.4* or *EMSLR* was related to poor OS or RFS in GSE37745. *p < 0.05; **p < 0.01; ***p < 0.001.

According to Liu et al. [1], the guide *RNAs GYS1*, *LRPPRC*, *NCKAP1*, *NDUFA11*, *NDUFS1*, *NUBPL*, *OXSM*, *RPN1*, *SLC3A2*, and *SLC7A1* were relatively fold changed between the glucose‐replete and ‐starved groups in the clear cell renal adenocarcinoma (ccRCC) cells, and these 10 DRGs were adopted for further analyses in this study. A total of 497 LUAD samples recruiting in the TCGA, database (https://www.cancer.gov/ccg/research/genome‐sequencing/tcga), of which 16876 lncRNAs were included. The clinical characteristics are shown in Table 1. The co‐expression analysis was screened between disulfidptosis‐related genes (DRGs) [1] and lncRNAs to identify the DRLs by co‐filter ≥ 0.4 and *p* < 0.001. Then we got 91 DRLs and most lncRNAs were related to the disulfidptosis‐related gene NDUFA11 (Figure 1A). After merging with survival data, univariable cox‐regression analysis accessed 11 candidate prognostic DRLs showed in the forest diagram (Figure 1B). Next, utilizing the methodology of LASSO regression analysis, a risk model was formulated based on candidate DRLs with the lowest error rates (Figure 1C,D). Subsequently, these candidate RNAs underwent thorough examination via multivariate Cox regression, ultimately culminating in the identification of five prognostic DRL signatures. The risk score was calculated as follows: (0.3223) × *AC092718.4* + (0.1775) × *AL365181.2* + (0.1567) × *AL606489.1* + (0.3231) × *EMSLR* + (−0.4627) × *ENTPD3‐AS1*. The correlation between the 10 DRGs and these five prognostic DRL signatures is shown in Figure 1E. We noted that some correlation tendency was not unique. In detail, *AC092718.4*, *AL365181.2*, and *EMSLR* were only positively correlated with the disulfidptosis genes, while *AL606489.1* and *ENTPD3‐AS1* were both positively and negatively correlated with the disulfidptosis genes. The aforementioned results implied that a complex network might be involved in regulating the lncRNAs and genes.

**TABLE 1:** The consistency test results of LUAD traits.

Covariates	Type	Total (%)	Train group (%)	Test group (%)	*p*
Age	≤ 65	239 (47.14%)	110 (43.31%)	129 (50.99%)	0.1371
	> 65	258 (50.89%)	137 (53.94%)	121 (47.83%)	
	Unknown	10 (1.97%)	7 (2.76%)	3 (1.19%)	
Gender	Female	272 (53.65%)	138 (54.33%)	134 (52.96%)	0.8263
	Male	235 (46.35%)	116 (45.67%)	119 (47.04%)	
Clinical stage	I	272 (53.65%)	128 (50.39%)	144 (56.92%)	0.4172
	II	120 (23.67%)	63 (24.8%)	57 (22.53%)	
	III	81 (15.98%)	45 (17.72%)	36 (14.23%)	
	IV	26 (5.13%)	15 (5.91%)	11 (4.35%)	
	Unknown	8 (1.58%)	3 (1.18%)	5 (1.98%)	
T Stage	T1	169 (33.33%)	77 (30.31%)	92 (36.36%)	0.1601
	T2	271 (53.45%)	137 (53.94%)	134 (52.96%)	
	T3	45 (8.88%)	29 (11.42%)	16 (6.32%)	
	T4	19 (3.75%)	10 (3.94%)	9 (3.56%)	
	Unknown	3 (0.59%)	1 (0.39%)	2 (0.79%)	
N Stage	N0	327 (64.5%)	161 (63.39%)	166 (65.61%)	0.3581
	N1	95 (18.74%)	47 (18.5%)	48 (18.97%)	
	N2	71 (14%)	40 (15.75%)	31 (12.25%)	
	N3	2 (0.39%)	0 (0%)	2 (0.79%)	
	Unknown	12 (2.37%)	6 (2.36%)	6 (2.37%)	
M Stage	M0	338 (66.67%)	164 (64.57%)	174 (68.77%)	0.3678
	M1	25 (4.93%)	15 (5.91%)	10 (3.95%)	
	Unknown	144 (28.4%)	75 (29.53%)	69 (27.27%)	

### 3.2 Validation of the Accuracy of the DRL Risk Model to Predict Patient Prognosis

The classification of the high‐ and low‐risk groups was executed through the use of the median risk score from the 5‐DRLs signature model. For validating the prognostic model, the whole cohort was randomly divided into a training group and a test group in a ratio of 1:1. Survival analysis results indicated that LUAD patients with high risk showed worse survival in both training (*p* < 0.001) and test (*p* = 0.004) groups (Figure 1F,G). The survival probabilities of the risk scores at 1, 3, and 5 year were denoted by an ROC value of 0.625, 0.666, and 0.666, respectively (Figure 1J). In the whole cohort, the Kaplan–Meier survival analysis curves effectively showcased that the low‐risk group had a better overall survival (*p* < 0.001, Figure 1H) and progression‐free survival (*p* = 0.019, Figure 1I) rate in contrast to their high‐risk counterparts. The Supplementary figure vividly presented the distribution of the risk score and survival status, both of which differ strikingly between the low‐ and high‐risk groups. It was revealed that patients with higher risk scores appeared shorter survival times and worse survival status (Figure S1A,C,E). Additionally, the expression of *AL365181.2*, *EMSLR*, and *AC092718.4* was increased, whereas the level of *ENTPD3‐AS1* was decreased in the high‐risk group, which might be a safeguarding element for LUAD patients (Figure 1K, Figure S1B,D).

Finally, datasets from GEO were recruited to explore the prognostic predicting value of the five signature DRLs in our prognostic model. However, because of the re‐annotation of lncRNAs in different microarrays and platforms, we did not observe data about lncRNA AL606489.1. Thus, we further analyzed the remaining four lncRNAs and performed the survival analysis of single lncRNA in GSE29013, GSE30219, GSE31210, and GSE37745 for *AC092718.4*, *AL365181.2*, *ENTPD3‐AS1*, and *EMSLR* (Figure 1L–O, Figure S1F–I). Interestingly, high expression of *AC092718.4*, *AL365181.2*, or *EMSLR* was an independent prognostic factor and related to worse OS, PFS, and RFS (recurrence‐free survival) in lung cancer patients. And lncRNA *AC092718.4* was the only shared prognostic factor in all the above four GSE datasets. Meanwhile, consistently with the prognostic model, *ENTPD3‐AS1* was a safeguarding element and high expression of *ENTPD3‐AS1* was related to better OS in the GSE30219 dataset (Figure 1M).

### 3.3 Validation of the Risk Model as an Independent Prognostic Factor

**FIGURE 2:**
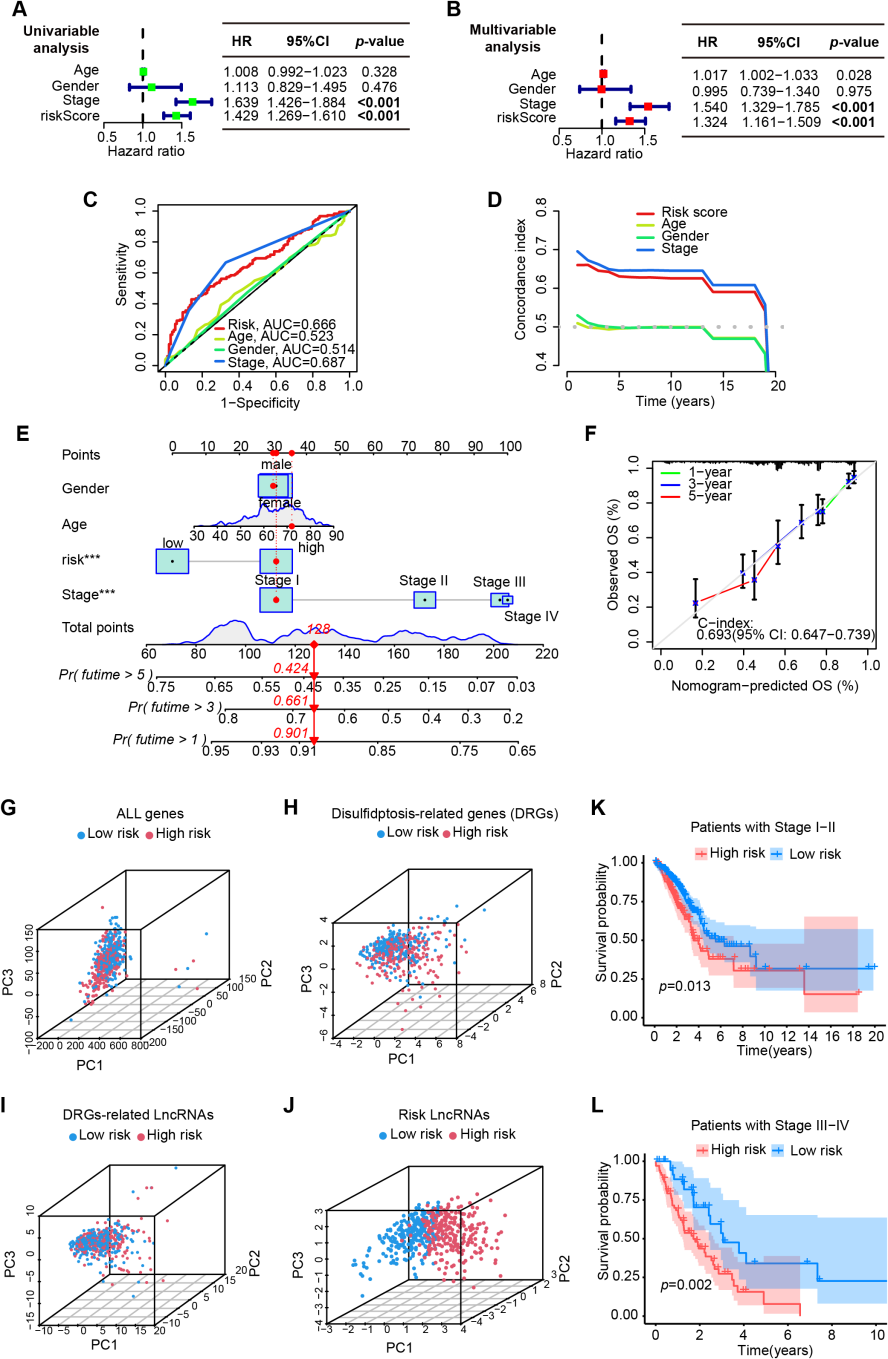
Validation of the risk model as an independent prognostic factor, construction of a predictive nomogram, and the group diagram of principal component analysis (PCA). Forest plots showing univariate (A) and multivariate (B) Cox regression analysis including the clinical information and risk score in LUAD. (C) Receiver operating characteristic (ROC) curves of the clinical information and risk score. (D) C‐index curves for risk score and clinical information, and verification of model‐predicted overall survival in patients with different clinical stages. (E) The nomogram model including the risk score, gender, age, and clinical stage predicted the probability of the 1‐, 3‐, and 5‐year OS. (F) The calibration curve for the OS nomogram. The expression of (G) all genes, (H) 10 disulfidptosis‐related genes (DRGs), (I) 91 disulfidptosis‐related lncRNAs, and (J) the five signature lncRNAs in low‐ and high‐risk groups. Kaplan–Meier curves for OS in patients with Stage I–II (K) and Stage III–IV (L).

In the whole TCGA‐LUAD cohort, the forest diagrams of univariable (Figure 2A) and multivariable (Figure 2B) Cox regression analyses indicated that the risk score could predict the survival independently as if the clinical stage (*p* < 0.001). ROC curves of clinical information suggested that the risk score just like the clinical stage had relatively good accuracy in predicting patient outcomes in terms of prognosis and survival (Figure 2C). C‐index curves also demonstrated that the risk‐scoring model had a good prediction power (Figure 2D). Together, the above outcomes revealed that the five DRL risk model possessed good stability in forecasting patient prognosis.

### 3.4 Construction of a Predictive Nomogram

Using risk score, gender, age, and clinical stage, we constructed a nomogram excepting to predict the OS of LUAD patients at 1, 3, and 5 year. A randomly picked patient sample got 128 points in total, whose OS incidence was 90.1%, 66.1%, and 42.4% at 1, 3, and 5 years after the diagnosis of LUAD, respectively (Figure 2E). Calibration plots of the nomogram showed the excellent agreement between observed and OS predicted by the nomogram at 1, 3, and 5 years (Figure 2C,F‐index = 0.693).

To judge the classification capability of the five DRLs risk model, the PCA was applied in all genes, DRGs, DRLs, and prognostic DRLs (Figure 2G–J). The results demonstrated that the prognostic lncRNAs owned a more effective distributing ability for low‐ and high‐groups (Figure 2J), indicating that the 5‐DRL signatures were an essential prognostic element for LUAD patients. Finally, the risk model also exhibited great prognostic value in both early (*p* = 0.013) and advanced‐stage (*p* = 0.002) LUAD patients (Figure 2K,L). Consistent with the previous results in all patients, better OS appeared in patients with lower risk scores either in the earlier stage or relatively advanced stage, which demonstrated once again that the risk model based on the five prognostic DRLs has a nice value in the prognostic prediction of LUAD patients.

### 3.5 Immune Characteristics Between Low and High Groups in LUAD Patients

**FIGURE 3:**
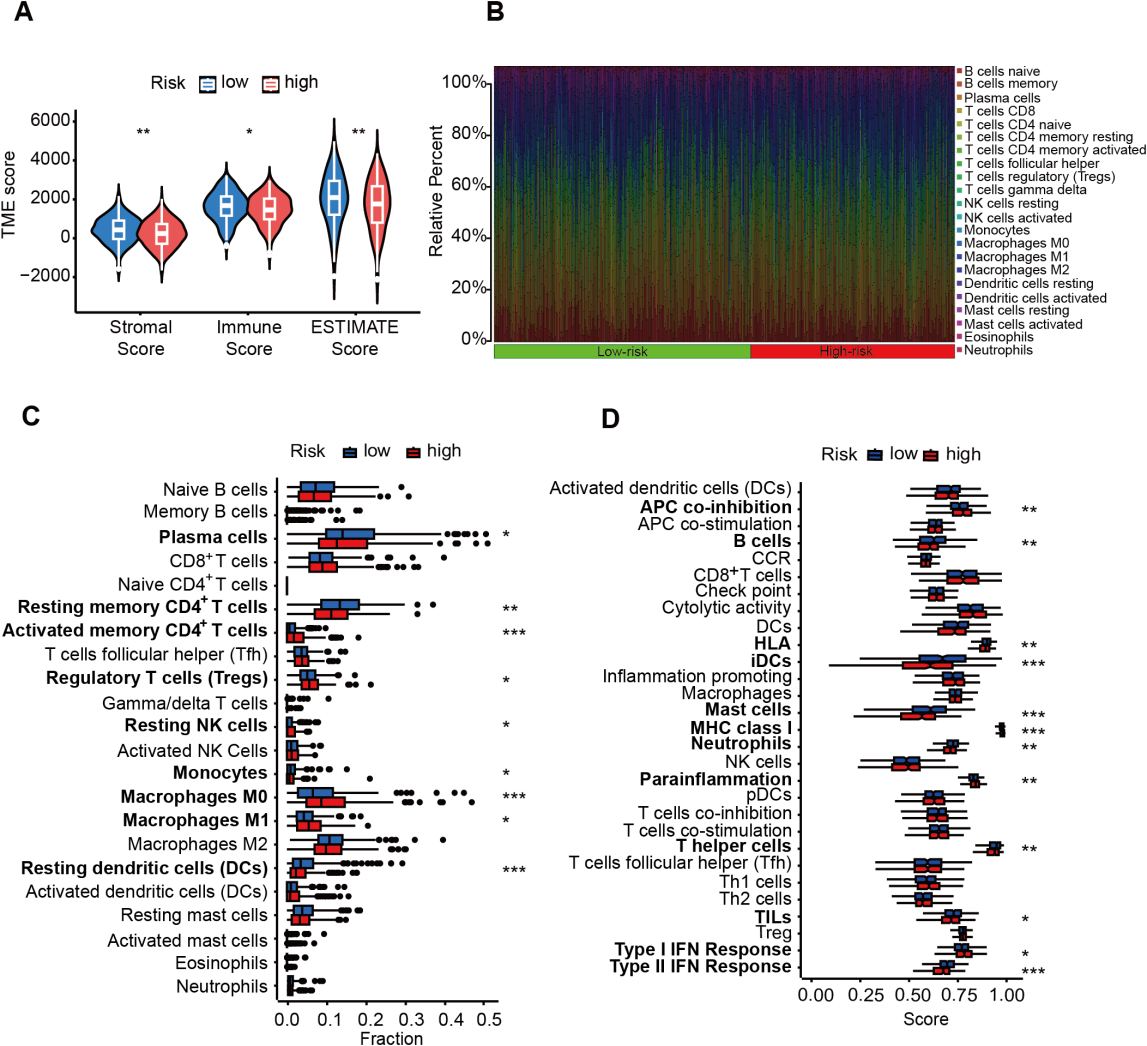
Different tumor environment (TME) between the low‐ and high‐risk group. (A) Comparison of TME score including stromal, immune, and ESTIMATE score between low‐ and high‐risk groups. (B) The relative distribution of tumor‐infiltrating immune cells was analyzed by CIBERSORT. (C) Differential abundance of 22 infiltrating immune cell types between low‐ and high‐risk groups. (D) Different 29 immune‐related functions in low‐ and high‐risk groups. *p < 0.05; **p < 0.01; ***p < 0.001.

To examine the tumor microenvironment (TME) score disparities between the low‐ and high‐risk groups, we employed the assistive services of ESTIMATE. The consequential findings disseminated that samples belonging to the low‐risk group garnered a considerably augmented stromal score, immune score, and ESTIMATE score (Figure 3A), which often represented better therapeutic outcomes and survival. Then, we adopted the CIBERSORT algorithm for a comprehensive insight into the distribution model of 22 tumor‐infiltrating immune cells between the low‐ and high‐risk groups (Figure 3B). The subjects with high‐risk score presented with lower infiltration abundance of plasma cells, resting memory CD4^+^ T cells, monocytes, resting DCs and higher abundance of activated memory CD4^+^ T cells, regulatory T cells (Tregs), resting NK cells, M0 and M1 macrophages (Figure 3C). Moreover, A positive correlation of risk score was found with the score of APC co‐inhibition, MHC class I, para‐inflammation, and type I IFN response, while a negative correlation existed with the score of B cells, HLA, mast cells, neutrophils, T helper cells, and type II IFN response (Figure 3D).

### 3.6 Tumor Mutational Burden Analysis

**FIGURE 4:**
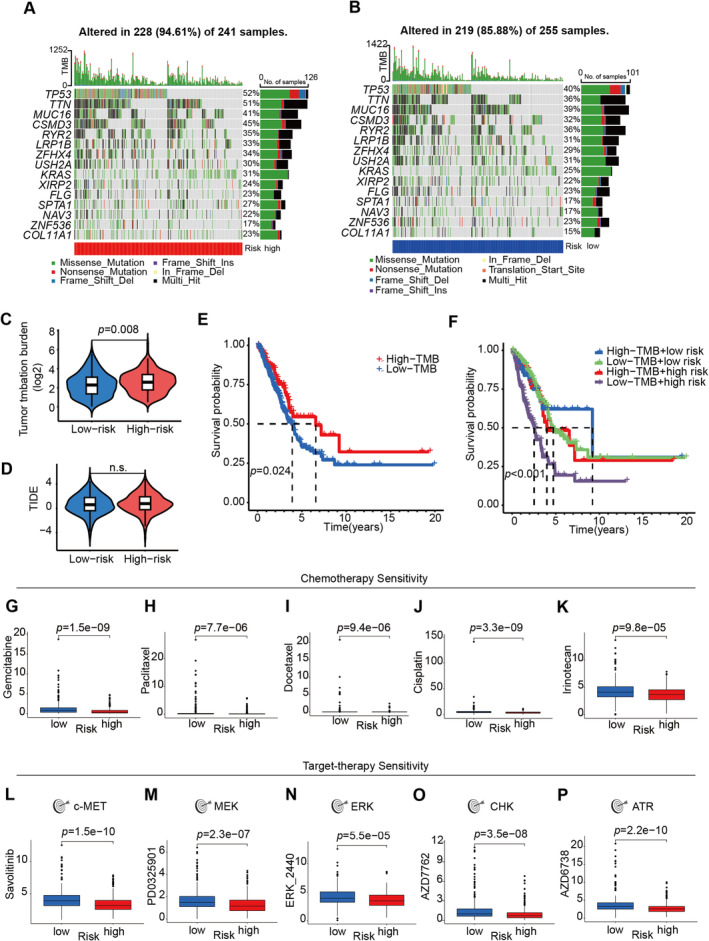
Tumor mutant burden (TMB) and drug sensitivity analyses. The waterfall chart of the frequently mutated genes in the (A) high‐ and (B) low‐risk groups. (C) Comparison of TMB between low‐ and high‐risk groups. (D) Comparison of tumor immune dysfunction and exclusion (TIDE) between low‐ and high‐risk groups. (E) Kaplan–Meier curves for OS of high‐ and low‐TMB groups. (F) Kaplan–Meier curves for OS of TMB and risk score. (G–K) The chemo‐drug sensitivity of gemcitabine, paclitaxel, docetaxel, cisplatin, and irinotecan was evaluated in low‐ and high‐risk groups. (L–P) The target therapy effect of c‐MET, MEK, ERK, CHK, and ATR inhibitors was estimated in low‐ and high‐risk groups. TIDE: http://tide.dfci.harvard.edu, n.s., no significance.

As there exists a direct correlation between tumor mutation burden (TMB) and both the clinical efficacy of immunotherapy and the survival prognosis of patients afflicted by cancer [11], it was analyzed in high‐ and low‐risk groups. About 94.61% of samples mutated in types of a missense mutation, nonsense mutation, frame‐shift deletion, frame‐shift insertion, or in‐frame deletion in the high‐risk group (Figure 4A), and the frequency of mutations in the low‐risk group was 85.88% (Figure 4B). A significant difference was noted in the TMB between the high‐risk and low‐risk groups (Figure 4C, *p* = 0.008). Although a nonsignificant difference showed in TIDE (Tumor immune dysfunction and exclusion) score between those two groups (Figure 4D), better overall survival was shown in patients in the high mutant burden group (*p* = 0.024, Figure 4E). Inspiringly, survival results based on the integration of TMB and risk score indicated that significant differences between all the four groups (*p* < 0.001), and patients with high‐TMB and the low‐risk score had the best survival probability (*p* < 0.001, Figure 4F).

### 3.7 Prediction of Drug Sensitivity

Finally, the possibility of assessing the pretreatment drug sensitivity was conducted based on the 5‐lncRNA prognostic model in LUAD. Patients with higher risk scores seemed more resistant to chemotherapy, such as gemcitabine, paclitaxel, docetaxel, cisplatin, and irinotecan (Figure 4G–K), which constitute the most commonly used chemotherapy protocols in LUAD patients with various stages. Compared to the small cell or squamous cell lung cancer patients, more LUAD patients could benefit from the target therapies including but not limited to EGFR TKIs, VEGFR inhibitors, or c‐MET inhibitors. Intriguingly, consistent with results from chemo‐drug analyses, LUAD patients in the high‐risk group were also resistant to c‐MET inhibitor Savolitinib, MEK, ERK, CHK, and ATR inhibitors (Figure 4L–P).

### 3.8 Functional Enrichment Analysis Based on the Disulfidptosis‐Related lncRNAs

**FIGURE 5:**
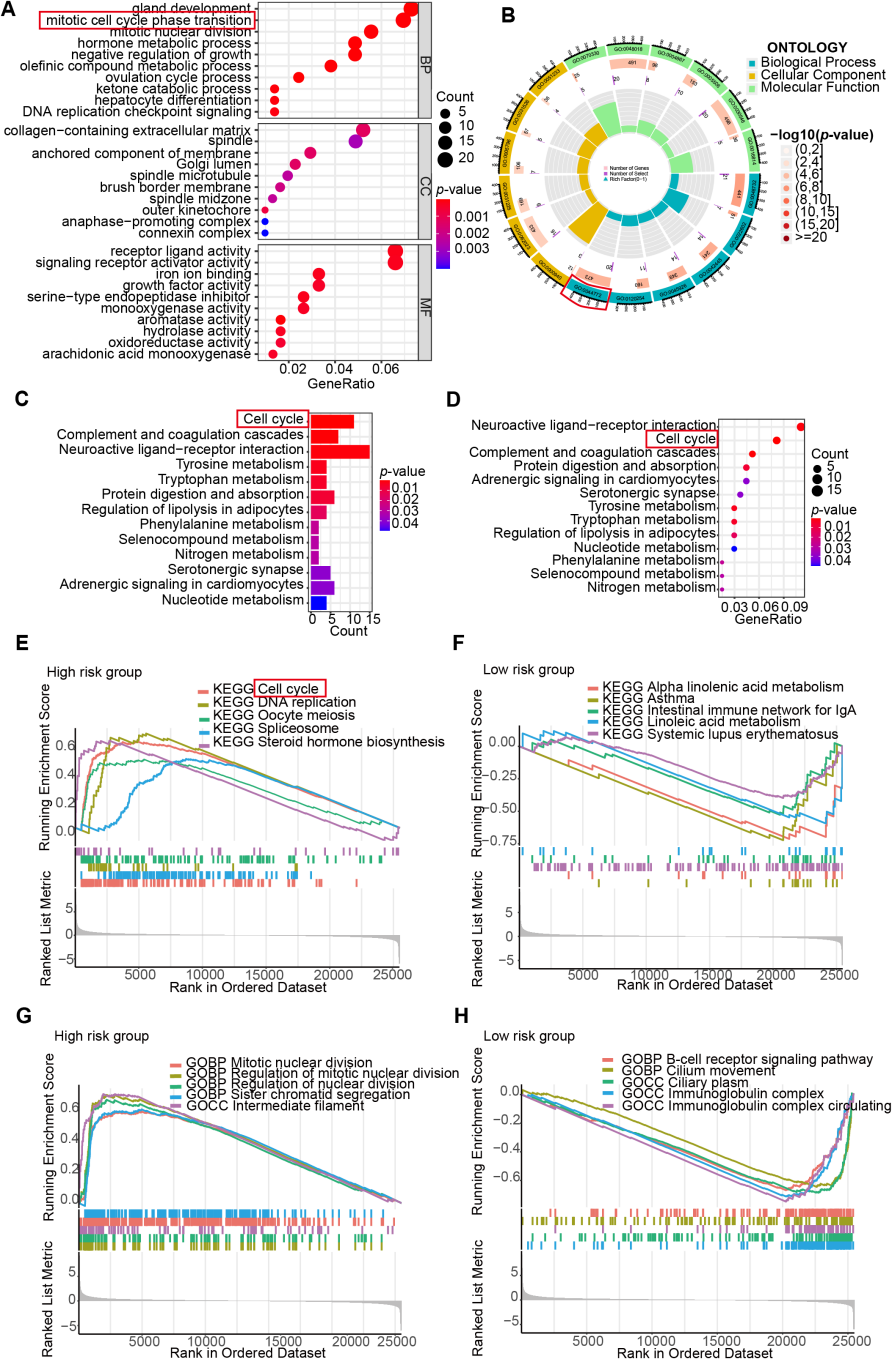
Enrichment analysis in high‐ and low‐risk groups based on the prognostic disulfidptosis‐related lncRNAs. (A) Bubble plots of the top 10 and (B) loop diagram of the top six GO‐enriched words based on the different expression genes between the low‐ and high‐risk groups. (C) Histogram and (D) bubble plot of the top 13 KEGG‐enriched words Enrichment analysis in high‐ and low‐risk groups. GSEA analysis based on the KEGG pathway database in (E) high‐ and (F) low‐risk groups. GSEA analysis based on the GO database in (G) high‐ and (H) low‐risk groups.

To gain a greater understanding of the possible biological processes and signaling pathways associated with the prognostic risk score signatures, we employed gene ontology (GO) enrichment as well as KEGG pathway analyses that were centered around the risk group based on the five prognostic DRLs. The outcomes of these analyses revealed that there was a notable enrichment of DEGs in gland development, collagen‐containing extracellular matrix, and receptor‐ligand activity (Figure 5A,B). The top three enriched signal pathways were cell cycle, complement and coagulation, and neuroactive ligand–receptor interaction (Figure 5C,D).

Additionally, we implemented GSEA in the high‐ and low‐risk groups, respectively. In GSEA‐KEGG analyses, the DEGs of the high‐risk group demonstrated significant enrichment in pathways about cell cycle, DNA replication, oocyte meiosis, spliceosome, and steroid hormone biosynthesis pathways (Figure 5E), whereas the DEGs in low‐risk group were mainly enriched pathways about alpha‐linolenic acid metabolism, asthma, the intestinal immune network for IgA production and systemic lupus erythematosus (Figure 5F). In GSEA‐GO analyses, the DEGs that characterized the high‐risk group exhibited notable concentration in processes relating the mitotic nuclear division, regulation of mitotic nuclear division, regulation of nuclear division, sister chromatid segregation and intermediate filament functions (Figure 5G), whereas the DEGs in low‐risk group were mainly concentrated in B‐cell receptor signaling pathway, cilium movement, ciliary plasm, immunoglobulin complex, and immunoglobulin complex circulating functions (Figure 5H).

### 3.9 LncRNA *AC092718* .*4* Promotes the Proliferation, Invasion, and Migration Abilities of LUAD Cell Lines

**FIGURE 6:**
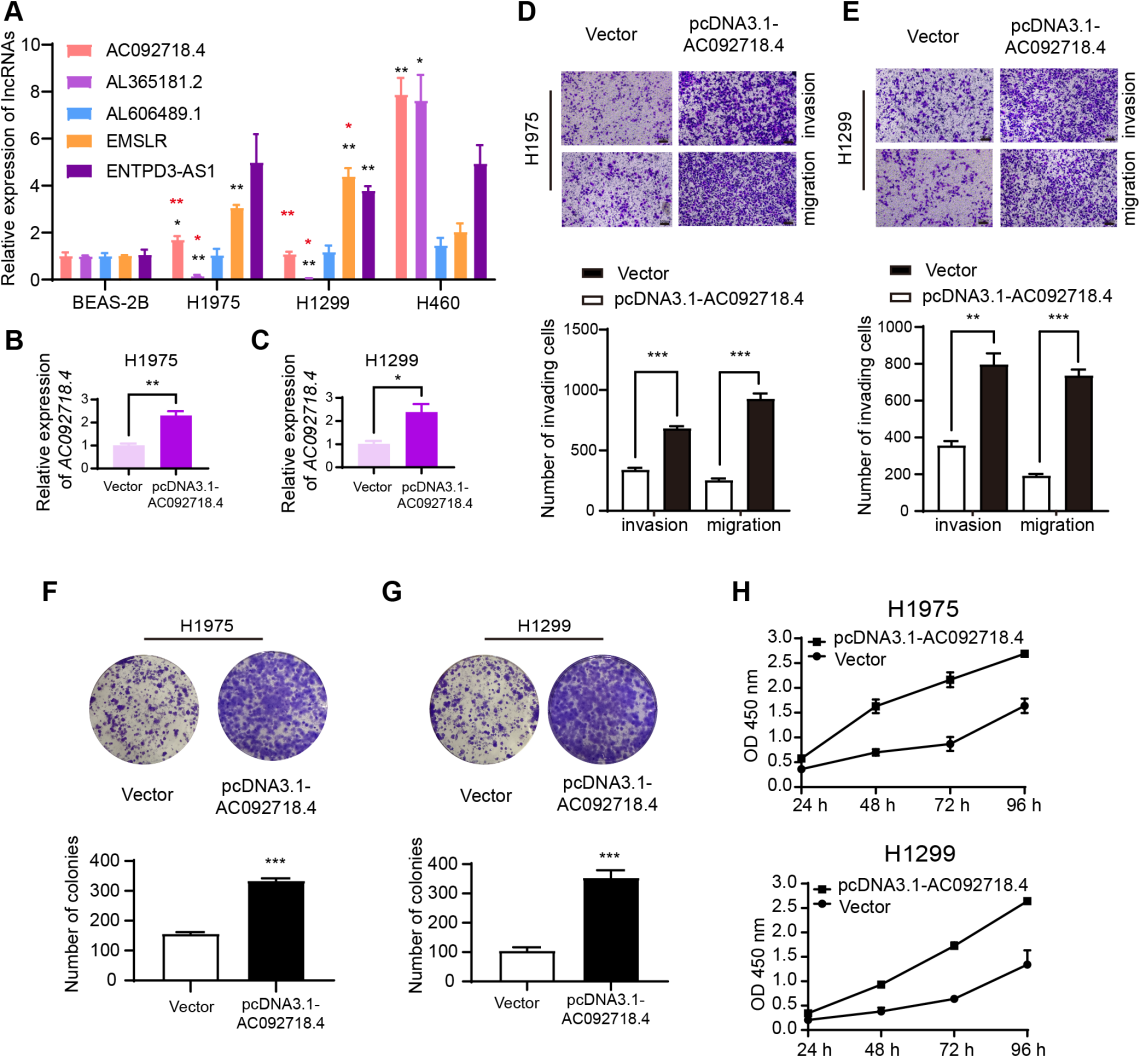
LncRNA *AC092718.4* promotes the proliferation, invasion, and migration abilities of LUAD cell lines. (A) The relative lncRNA expressions were analyzed by quantitative real‐time PCR (qRT‐PCR). The relative expression of *AC092718.4* increased by transient transfections in H1975 (B) and H1299 (C) cells. (D) Upregulation of *AC092718.4* significantly accelerated the invasion (up panel) and migration (down panel) abilities of H1975 cells. (E) Upregulation of *AC092718.4* accelerated the invasion (up panel) and migration (down panel) abilities of H1299 cells. (F) Colony‐forming assays indicated that high expression of *AC092718.4* promoted the colony ability of H1975 (F) and H1299 (G) cells. (H) An increased absorbance at 450 nm was observed in *AC092718.4* overexpressed H1975 (up diagram) and H1299 (down diagram) cells. *p < 0.05; **p < 0.01; ***p < 0.001.

The relative expressions of the five signature DRLs were examined by qRT‐PCR. Compared to benign BEAS‐2B cells, most signature DRLs were overexpressed in lung cancer cell lines (Figure 6A, black asterisks). The expressions of *AC092718.4* and *AL365181.2* were decreased in H1975 and H1299 cells compared to H460 cells known to be highly aggressive (Figure 6A, red asterisks) [12]. Combined with the aforementioned results that high expression of *AC092718.4* was a poor prognosis factor in all four GSE datasets (Figure 1L–O), lncRNA *AC092718.4* was chosen to further understand the involved functions and underlying mechanisms in LUAD cell lines. After transfection with pcDNA3.1‐AC092718.4, the expressions of *AC092718.4* were significantly increased in H1975 (Figure 6B) and H1299 (Figure 6C). And results of transwell assays indicated that overexpression of *AC092718.4* enhanced the invasion and migration abilities of H1975 (Figure 6D) and H1299 cells (Figure 6E). Moreover, we applied colony‐forming and CCK8 assays to explore the role of *AC092718.4* in tumor proliferation. As the outcomes show, the upregulation of *AC092718.4* contributed to the colony formation of LUAD cells (Figure 6F,G). Consistently, we observed that the absorbance of OD 450 nm was enhanced by the overexpression of *AC092718.4*, indicating an improved tumor proliferative capacity in pcDNA3.1‐AC092718.4‐transfected cells (Figure 6H). Experiments including but not limited to *AC092718.4* downregulation, bulk, and single‐cell RNA‐sequencing will be completed in our future study.

## 4 Discussion

The rapidly advancing research on programmed cell death has expanded the outlook on its potential applications in cancer diagnosis, prognosis, and treatment [13, 14, 15]. Recent research suggested that a unique form of programmed cell death, disulfidptosis, might hold great promise as a therapeutic target for combating cancer [1]. Meanwhile, it has been demonstrated that lncRNAs serve a pivotal function in lung cancers by regulating the oncogenes, tumor suppressor genes, micro‐RNAs, epigenetics, signaling pathways, and immune functions in lung cancer [16, 17, 18, 19, 20, 21]. While the function of disulfidptosis‐related lncRNAs in LUAD remains unclear. In this study, we explored the lncRNAs related to disulfidptosis in LUAD for the first time and constructed a 5‐DRL risk score model. Intriguingly, the risk model has demonstrated impressive efficacy in accurately identifying high‐risk patients who are susceptible to unfavorable overall and progression‐free survival and was an independent prognostic factor for the LUAD patients. Further function analyses showed that pathways related to cell cycle, IgA production, and cell division were involved, and the DRLs were also shown to be relevant to the immune microenvironment and the drug sensitivities of LUAD. To the best of our knowledge, the character of the disulfidptosis‐related lncRNA in LUAD has not been examined before, and our study could be a good extension of the existing research.

The bioinformatics analysis demonstrated that only five disulfidptosis‐related lncRNAs were identified as prognostic signatures, which were four oncogenes: *AC092718.4*, *AL365181.2*, *AL606489.1*, *EMSLR*, and the antioncogene *ENTPD3‐AS1*. The roles of these DRLs were further confirmed in GSE datasets, and lncRNA *AC092718.4* was related to poor prognosis of patients in GSE29013, GSE30219, GSE31210, and GSE37745. In the field of oncology and medicine, nomograms are a commonly used tool for prognostic prediction [22]. We developed a nomogram incorporating the DRL‐related risk score, gender, age, and clinical stages. The nomogram's ability to predict 1‐, 3‐, and 5‐year OS of LUAD patients was found to be effective, which highlighted its potential in promoting individualized treatment for LUAD patients.

To date, the advent of the tumor microenvironment and TIL might grasp the potential to overcome the current therapeutic limitations in non‐small cell lung cancer (NSCLC). Types of the infiltrating immune cells in the tumor microenvironment (TME) showed great influence on the clinical outcomes across cancer types [23]. Stromal cells were widely posited to play vital roles in the progression of tumors and in conferring resistance to drugs employed for cancer treatment [24, 25]. Studies indicated that worse OS showed in LUAD patients with a lower immune score, stromal score, or estimate scores [26]. We speculated that the prognostic DRLs might affect the LUAD patients' survival via regulating the tumor microenvironments, and applied the ESTIMATE and CIBERSORT algorithms for discerning the variance in the composition of tumor‐infiltrating immune cells in the low‐ and high‐risk groups. The results showed that the stromal, immune, and ESTIMATE scores had a notable surge in better‐surviving patients with low‐risk scores. The portion difference in 22 infiltrating immune cell types was significant between the low‐risk and high‐risk groups. The overexpression of prognostic onco‐lncRNAs might contribute to CD4^+^ T cell depletion via increased APC co‐inhibition, which primarily mediates anti‐tumor immunity by providing help for CD8^+^ cells [27, 28]. These analyses further added credibility to our risk model and implied that the five prognostic lncRNAs might implement their functions through regulating tumor microenvironment in LUAD. The above results highlighted the ability of our lncRNA signature not only to predict survival outcomes but also to reflect immune cell infiltration levels.

Previous research has shown that lung cancer patients could gain significant advantages from PD‐1/PD‐L1 inhibitors and that TMB is associated with outcomes with immunotherapies, but the correlation is still controversial [29]. In our study, patients with higher TMB showed a better survival probability. Unexpectedly, poor prognostic patients with high‐risk scores showed higher TMB scores which were usually thought related to better therapeutic outcomes. That might be explained by the complexity of immune‐related regulators, and the comprehensive multifactor analysis was urgent to predict the patients' survival when involving immunotherapies. In the present study, we used the combinatory analyses of TMB score and risk score trying to forecast the outcomes of immunotherapies more precisely. It was noteworthy to observe that the subgroup of patients classified as high risk and possessing low TMB exhibited the poorest survival outcomes when compared to the entire cohort. This observation suggested that the reduced frequency of mutant genes within the high‐risk group contributes to their unfavorable prognosis. In addition, we used the oncoPredict R package and the GDSC database to calculate the sensitivity of antitumor drugs, and the patients with high‐risk scores were more resistant toward the standard chemotherapeutic agents for LUAD as well as the targeted drugs that are already in clinical practice or clinical trials. Our findings have been quite promising in the identification of prognostic biomarkers that could aid in better clinical decision‐making for the treatment of diverse immune subtypes. This may ultimately translate to improved therapeutic outcomes for patients who require multiple therapies.

Then our analysis focused on understanding the biological mechanisms underlying the risk groups identified by the five lncRNA signatures. Previous research reported that the expression of *AC092718.4* contributed to the tumor progression by affecting the CD8^+^T cell infiltration in breast cancer [30], and was a tumor promoter in LUAD without clarifying the underlying mechanisms [31]. Fischer et al. displayed that the p53‐p21‐DREAM/RB signaling could regulate the lncRNA *AC092718.4* expression via the targeted gene *CENPN* directly hosting to its 3′UTR in human colorectal carcinoma cell line HCT116 [32]. Additional research is required to thoroughly elucidate the molecular mechanisms of *AC092718.4* in LUAD. Thus, we carried out several typical cell biology experiments and found that overexpressed *AC092718.4* promoted the invasion, migration, and proliferation abilities of H1975 and H1299 LUAD cell lines in vitro. To the best of our knowledge, it was the first time that the functions of *AC092718.4* were confirmed by biological experiments in LUAD cells. Functional analyses in our study also showed that the biological cell cycle transition process and cell cycle pathway were involved in the patient group with high expression of prognostic onco‐lncRNAs such as *AC092718.4*. It is reasonable to assume that *AC092718.4* might regulate the LUAD cells' biological behaviors via pathways relating to the cell cycle. While more experiments such as bulk and single‐cell RNA sequencing are necessary in our following study to understand the relevant pathways and molecular mechanisms involved in *AC092718.4*.

Similar to the present study, research reported that lncRNA *AL365181.2* might affect the immune statuses of cancer patients [33, 34], without clarifying the underlying molecular mechanisms. Likewise, lncRNA *AL606489.1* was reported to act as an oncogene, and its overexpression was deleterious to the survival of patients with lung, live, or cervical cancer [35, 36, 37]. The functions of *AL606489.1* participated in various biological processes including necroptosis, pyroptosis, ferroptosis, cuproptosis, autophagy, and N6‐methyladenosine [38, 39, 40, 41, 42], while the potential mechanisms are not yet clear. The overexpressed *EMSLR* was a signature of poor prognosis in endometrial carcinoma and muscle‐invasive bladder cancer [43, 44]. Researchers indicated that *EMSLR*, one of the prognostic signatures in our risk model, could lead to G1 block, impeded S phase progression, and inhibited the tumor‐related phenotypes when it was depleted in A549 cells [45, 46]. The c‐MYC/E2F1 signal transduction pathway showed the potential of repressing the promoter activity of *lncPRESS1* in the function of *EMSLR* for the cell cycle. Differently from the other four tumorigenic DRL signatures, lncRNA *ENTPD3‐AS1* was a tumor suppressor gene within the scope of the current study and previous research scope [47]. The expression of *ENTPD3‐AS1* was enhanced by the G > A mutation of rs67311347 forming a binding motif of ZNF8. Then *ENTPD3‐AS1* could interact with *miR‐155‐5p* and upregulated the expression of HIF‐1α, inhibiting cell proliferation and tumor suppression In RCC [47].

In the current study, we did a comprehensive exploration and validation of the prognostic signature of the disulfidptosis‐related lncRNAs using TCGA and GSE datasets for the first time, which might be used clinically in subgrouping LUAD patients with different survival tendencies, immune infiltration levels, tumor mutation burdens, and therapy sensitivities in clinical practice. The biological function of lncRNA *AC092718.4* has been initially explored in LUAD cell lines. However, our research exhibited certain constraints and limitations. Further relevant studies in pan‐cancer needed to be fulfilled, meanwhile high‐throughput RNA‐sequencing analysis including all 5‐lncRNA was needed to verify our prognostic model using in‐house LUAD patient data.

## 5 Conclusion

We constructed a disulfidptosis‐related risk model that could not only enable survival prediction for LUAD patients for the first time but also could help physicians make clinical decisions by forecasting the TME, TMB, and drug sensitivity levels. The model comprising five disulfidptosis‐related lncRNAs might be a clinically promising tool for survival and outcome predictions for LUAD patients.

## Author Contributions

**Xin Nie:** data curation (lead), formal analysis (lead), funding acquisition (lead), writing – original draft (lead). **Hong Ge:** project administration (equal). **Kongming Wu:** project administration (equal), validation (lead). **Ru Liu:** methodology (equal). **Chunyu He:** methodology (equal).

## Conflicts of Interest

The authors declare no conflicts of interest.

## Supporting information


**Figure S1.** Validation of the accuracy of the DRL risk model to group LUAD patients. Distribution of risk score and survival status in training group (A), test group (C), and whole cohort (E). The expression of prognostic DRLs in low‐ and high‐risk groups in training (B) and test group (D). (F) The effect of the expression of *AL365181.2*, *EMSLR*, or *ENTPD3‐AS1* on the patients’ survival in GSE29013. (G) The effect of the expression of *EMSLR* or *ENTPD3‐AS1* on the patients’ survival in GSE30219. (H) The effect of the expression of *AL365181.2*, *EMSLR*, or *ENTPD3‐AS1* on the patients’ survival in GSE31210. (I) The effect of the expression of *AL365181.2*, *ENTPD3‐AS1*, or *EMSLR*, on the patients’ survival in GSE37745.

## Data Availability

The data that support the findings of this study are openly available in TCGA at https://portal.gdc.cancer.gov/ and GEO at https://www.ncbi.nlm.nih.gov/geo/.
