# Supplementary material for: Unlocking the Potential of Disulfidptosis‐Related LncRNAs in Lung Adenocarcinoma: A Promising Prognostic LncRNA Model for Survival and Immunotherapy Prediction
Source: Cancer Med. 2024 Oct 21;13(20):e70337. doi: 10.1002/cam4.70337 (PMC11492340; doi:10.1002/cam4.70337)
Supplement: Supplementary file 1 — Figure S1. Validation of the accuracy of the DRL risk model to group LUAD patients. Distribution of risk score and survival status in training group (A), test group (C), and whole cohort (E). The expression of prognostic DRLs in low‐ and high‐risk groups in training (B) and test group (D). (F) The effect of the expression of AL365181.2, EMSLR, or ENTPD3‐AS1 on the patients’ survival in GSE29013. (G) The effect of the expression of EMSLR or ENTPD3‐AS1 on the patients’ survival in GSE30219. (H) The effect of the expression of AL365181.2, EMSLR, or ENTPD3‐AS1 on the patients’ survival in GSE31210. (I) The effect of the expression of AL365181.2, ENTPD3‐AS1, or EMSLR, on the patients’ survival in GSE37745. [file CAM4-13-e70337-s001.docx]

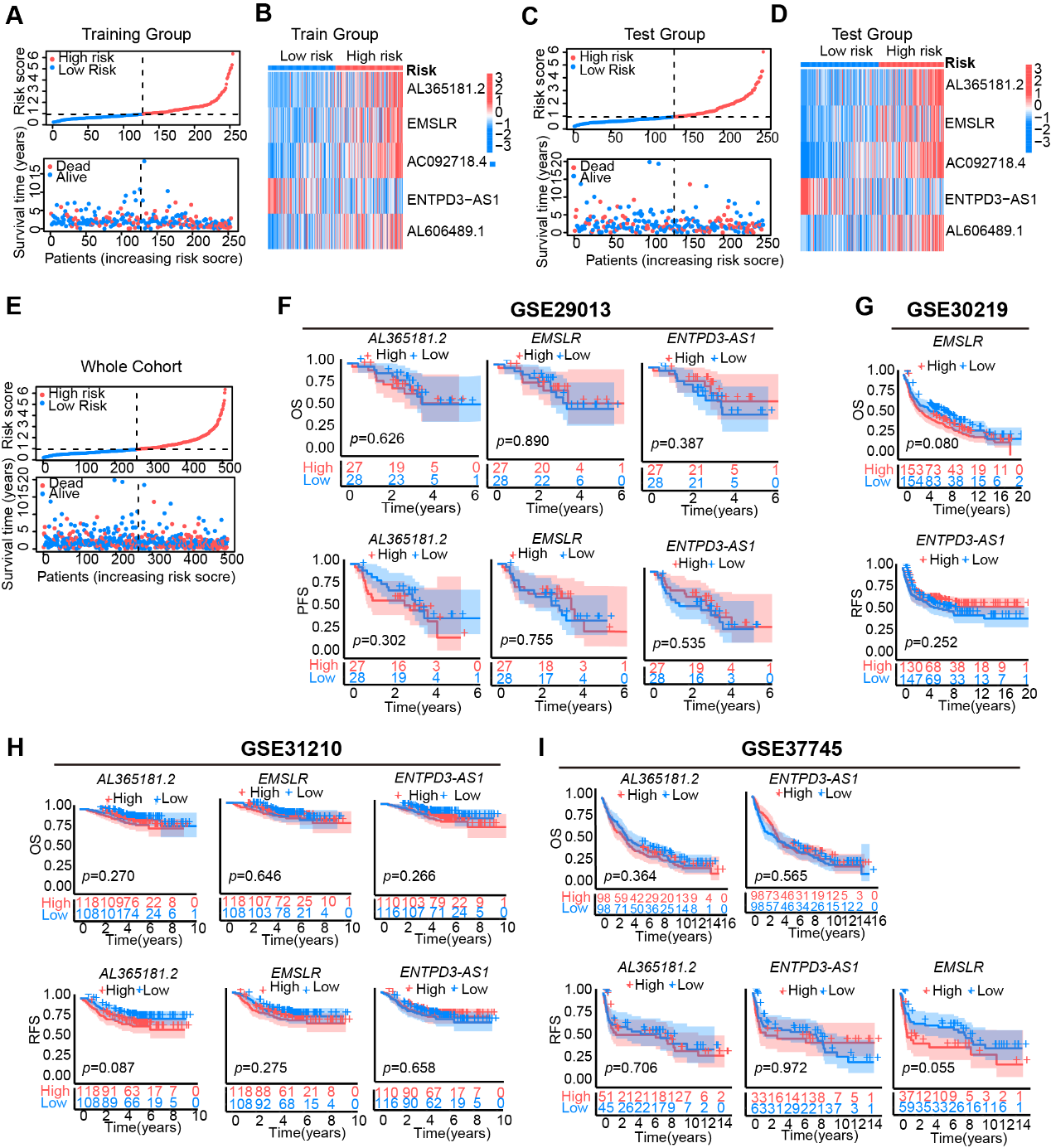


Supplementary Figure 1 Validation of the accuracy of the DRL risk model to group LUAD patients. Distribution of risk score and survival status in training group (A), test group (C), and whole cohort (E). The expression of prognostic DRLs in low- and high-risk groups in training (B) and test group (D). (F) The effect of the expression of *AL365181.2, EMSLR,* or *ENTPD3-AS1* on the patients’ survival in GSE29013. (G) The effect of the expression of *EMSLR,* or *ENTPD3-AS1* on the patients’ survival in GSE30219. (H) The effect of the expression of *AL365181.2, EMSLR,* or *ENTPD3-AS1* on the patients’ survival in GSE31210. (I) The effect of the expression of *AL365181.2, ENTPD3-AS1,* or *EMSLR,* on the patients’ survival in GSE37745.
